# Erratum for Petch et al., “Feline Leukemia Virus Frequently Spills Over from Domestic Cats to North American Pumas”

**DOI:** 10.1128/jvi.02169-24

**Published:** 2025-01-16

**Authors:** Raegan J. Petch, Roderick B. Gagne, Elliott Chiu, Clara Mankowski, Jaime Rudd, Melody Roelke-Parker, T. Winston Vickers, Kenneth A. Logan, Mathew Alldredge, Deana Clifford, Mark W. Cunningham, Dave Onorato, Sue VandeWoude

## ERRATUM

Volume 96, no. 23, e01201-22, 2022, https://doi.org/10.1128/jvi.01201-22. Page 12: Table 3, last row, the 500 bp reverse primer sequence should read “GAGTCCATGTTACAGCCC.”

